# Smartphone Addiction and Its Associated Factors Among Tehran University Students

**DOI:** 10.1192/bjo.2022.165

**Published:** 2022-06-20

**Authors:** Ali Kheradmand, Elhamsadat Amirlatifi

**Affiliations:** 1 Taleghani Hospital Research Development Committee, School of Medicine, Shahid Beheshti University of Medical Sciences, Tehran, Iran, Islamic Republic of Iran; 2 Northamptonshire Healthcare NHS Foundation Trust, Memory Assessment Service and Community Mental Health Team for Older Adults, Rushden Resource Centre, Rushden, United Kingdom

## Abstract

**Aims:**

Smartphone addiction is a new concern due to its progressive global usage. Since this phenomenon occurs in adolescents and young people, especially in students, causing many problems in interpersonal relationships, occupational and educational performances, evaluation of smartphone addiction in this population seems to be necessary. Accordingly, this study aimed to examine the prevalence of smartphone addiction in Tehran university students for determining the risk factors associated with this issue.

**Methods:**

This analytical cross-sectional survey was carried out on university students in Tehran between 2016–2018. A study sample of 382 students from various faculties of Tehran universities was chosen by random multi-stage cluster sampling. The participants simultaneously completed a researcher-made questionnaire on demographic characteristics and risk factors, the Smartphone Addiction Scale (SAS), and Young's Internet Addiction Test (IAT). After checking the smartphone addiction questionnaire, smartphone-addicted individuals were identified, and a comparison with the non-addicted group was performed in terms of risk factors.

In this study, the participants were given written consent forms. Questionnaires were anonymous and the information was kept confidential. This study was registered at the Ethics Committee of Shahid Beheshti University of Medical Sciences with a code of 1395,309.

**Results:**

Our findings indicated that the frequency of smartphone addiction was 28.8%. The frequency of smartphone addiction in women (32.5%) was higher than that in men (23%) (p = 0.04). The highest incidence of smartphone addiction occurred in the age range of less than 20 while the lowest was found above the age of 40. (P = 0.001). The prevalence of smartphone addiction in the single population was 34.1% Vs 16.1% in married. (P = 0.000) The most prominent educational field of smartphone addicts was technical and engineering. (P = 0.007). Smartphone addiction was significantly more in internet service and social networking users. (P = 0.025) There was a significant relationship between the history of psychiatric illness and smartphone addiction. (P = 0.035) The most common psychiatric diseases were found to be obsessive-compulsive disorder (41.7%), followed by anxiety disorders. (33.3%)

**Conclusion:**

Smartphone addiction has a significant frequency among university students in Tehran, associated with access to internet services and social networking. It was more common in women, single adults, and younger ages. There was a significant relationship between the history of psychiatric illness and smartphone addiction. The highest frequency of psychiatric illnesses in the addiction group was obsessive-compulsive disorder and anxiety disorders. No relationship was found between smartphone addiction and history of substance use, as well as smoking and alcohol.

